# Crisis, justice, and managing the appetite for risk

**DOI:** 10.1007/s41020-022-00165-w

**Published:** 2022-06-14

**Authors:** Francine Rochford

**Affiliations:** La Trobe University, Melbourne, VIC Australia

**Keywords:** Human rights, COVID-19, Separation of powers, Discursive framing

## Abstract

The phrase ‘never let a good crisis go to waste’ is often (mis)attributed to Winston Churchill. It expresses the common perception that the sentiments evoked by crisis can be used to manipulate power relations and strategically reposition influence. Although crises can arise from tangible, objectively catastrophic external events, governmental responses to crises are accompanied by processes of framing—construction, interpretation, and communication to the community subject to governance. The framing and management of crises can contribute to the expansion of regulatory scope. Moreover, the process of scaffolding regulatory legitimacy during times of crisis involves deployment and amplification of techniques of governance such as the use of data and expert knowledge, risk management, responsibilisation, and dispersal (or non-dispersal) of funds. In political systems that have adopted neoliberalist forms, these techniques of governance cascade from state-administered functions, such as policing, health, armed forces, and emergency services, to local communities, employers, and consumers. In this way we find responsibility devolved and detached from political decision-making and, more importantly, from democratic legitimacy. Dispersed governing mechanisms steer individual practices towards certain ends so that, rather than suffering the removal of the capacity for decision-making, individuals willingly abandon it. This article explores the intersection between regulation and justice and the methods of framing crises to legitimise governance actions where those actions constrain human rights and justice claims. Analysing Australian state and federal governmental and non-governmental actions during the COVID pandemic, it will use a case study method to assert that performative compliance activity is amongst the suite of sophisticated techniques to legitimise decisions made in circumstances of crisis, even when those decisions cross traditional normative boundaries, implicitly diminishing claims to legitimacy based on democratic discourse. This article will focus on two events: the decision to ‘lock down’ community housing towers, and the decision to arrest and charge with incitement a pregnant woman for starting a Facebook post to encourage breach of lockdown restrictions. Both decisions prompted expressions of concern by civil rights groups and lawyers that human rights had been breached.

## Introduction

The health impacts of the COVID-19 pandemic have precipitated a suite of executive responses, enabled by legislative intervention, in most jurisdictions. Many responses have involved a suspension of certain rights and freedoms,^1^ prompting the assessment that ‘the condition of democracy and human rights has grown worse in 80 countries.’^2^ Since the detection of COVID-19 in a patient in Australia in January 2020, Australian states have implemented significant restrictions on movement and assembly and have enforced detention for the purpose of quarantine. The Victoria State Government sought (and obtained) additional power to declare pandemics and increase capacity to enforce public health orders, vesting additional responsibilities in the Minister of Health.^3^ The abrogation of established human rights has raised legitimate questions about the parameters of human rights and their elasticity in the face of emergency. Moreover, it demonstrates a degree of narrative flexibility in definitions of necessity as they attach to questions of public health. These issues are not confined to Australia, which lacks a Bill of Rights, and supplemental questions arise as to the resilience of any rights concept mediated by language. Conversely, rights language has been extended to assert rights, inter alia, to remain unvaccinated, to move between states, not to wear masks, and to attend parties.^4^

This article will consider the responses of Australian jurisdictions to the health emergency. It will articulate the capacities of the state to deal with health emergencies, which were certainly anticipated at the time of the creation of the Australian polity. Noting that social and regulatory life is subject to increasing ‘juridification’,^5^ it will elaborate on now common techniques of governance that frame and reframe narratives of crisis, justifying regulatory actions that would otherwise be considered a breach of human rights, and the aspects of crisis that demonstrate that the diminution of rights pre-dated the pandemic, and it is only the public appetite for the extent of that diminution that is now being tested.

The article will commence by outlining the Australian legal landscape protecting human rights, first outlining the minimal human rights protections articulated in the Australian Constitution^6^ (and how human rights protections align with quarantine and other emergency powers). It will then introduce the federal human rights statutory protections and will briefly consider the parallel state protections for human rights, focussing on the Victorian situation. This will demonstrate that, despite the lack of an explicit Bill of Rights in the Constitution, there are legislative and common law protections for established civil, economic, and political rights. These protections include freedom of political speech, freedom of association, the right to work, and the overarching maintenance of the separation of powers and the rule of law. In common with those countries with constitutionally protected rights and freedoms, some rights give way to countervailing obligations in times of pandemic emergencies. As in all law, the balancing of countervailing necessities is mediated by open linguistic forms, such as ‘reasonable’, ‘proportionate’, ‘reasonable necessity’, and ‘reasonably appropriate and adapted’. Accordingly, the rights landscape is vulnerable to discursive framing and techniques of manipulation.

To demonstrate explicit clashes of established rights and pandemic measures, this article will take two case studies from the Victorian State context. The summary lockdown of public housing towers in Melbourne illustrates the collision between the rights to freedom of movement and to employment, and in the context of the vulnerable communities involved becomes an even more marked departure from established liberties. This was followed by a lengthy lockdown of the entire state, including curfews^7^ and regulation of exercise,^8^ employment, and travel.^9^ The suppression of these activities was scaffolded by the utilisation of state police in roadblocks, home visits to check on movements, and stop and search. Anticipating the exercise of the civil right to protest against these draconian measures, police power was also exerted against persons rejecting the basis for or implementation of these techniques. The arrest of a Victorian woman for posting on Facebook an invitation to protest against lockdowns^10^ is a clear derogation from an established (if implied) freedom of political communication and the right to freedom of association.^11^

The discursive framing of these events, and other pandemic measures, became a process of ongoing legitimation in the face of changing perceptions of risk and reliability of the measures taken. The article will analyse the narrative frameworks within which the regulatory actions took place. It will conclude that the modern forms of governance in crisis, utilising multiple and linked media formats, create a space in which expert narrative framing and use of discursive techniques can effectively override objectively legitimate concerns about human rights protections, effectively narrowing human rights protections to a circumscribed civil space bounded by expert assertions of risk.

## The Australian legal landscape

Democratic nations typically look to the legal infrastructure for protection of human rights. It is common to exhort the necessity for constitutional acknowledgements of fundamental rights, but protection may also arise from less fundamental legislation. Moreover, the common law provides significant protections. Pandemic situations, like war emergencies, are acknowledged justifications for departure from certain civil and economic rights. However, established jurisprudence denotes these departures as exceptional. For instance, in the case of the defence power, a court ruling on a challenge to legislation would have to be given evidence of the fact of hostilities, or alternatively it would have to be a matter of judicial notice. Once the Australian federal defence power is activated it enables the federal government to make valid enactments that deny certain civil liberties. However, this legislation would not be valid in peacetime.^12^ The federal government is not able to simply ‘recite itself into power’ by merely stating that a war situation is extant.^13^ Australian response to disease risk is, similarly, often phrased in terms of war: a ‘language of defence of nation—resistance, protection, invasion and immigration’^14^ characterises the national conversation about quarantine. The quarantine power, contained in s 51(ix) of the Constitution, is supported by s 69 which requires the states to transfer the public service departments of quarantine to the Commonwealth, although states continued to exercise quarantine powers between states. In preventing human movement, banning public gatherings, requiring isolation of infected individuals, and mandating masks or immunisation, Australian quarantine legislation routinely acknowledges the valid departure from certain civil liberties to prevent disease transmission, at both state and federal levels.^15^ It is clear that the Australian federation anticipates situations in which freedoms are suspended in order to protect other values, such as human life or the life of the nation. However, the existence of multiple levels of government muddies the legal landscape in terms of: (*a*) where human rights protections originate; (*b*) which protections override others; and (*c*) when those protections can be suspended for good reason. This section will briefly consider the Australian legal landscape, to provide a background for the Victorian use of power and the existing constraints against that power.

The Constitution establishes a federal system of government, perpetuates the separation of powers between the executive, legislature, and judiciary,^16^ and divides enumerated legislative powers between the federal and state levels of government (although the federal balance is prone to centralisation).^17^ It also, in s 109, establishes a mechanism to resolve conflict between federal and state legislation: in the event of inconsistency between federal and state legislation, the federal statute prevails to the extent of the inconsistency, creating a protection for the individual that they will not be subjected to conflicting laws.^18^ The High Court is established and constitutionally empowered to resolve disputes arising from constitutional interpretation.^19^

The Constitution is famously unencumbered with a Bill of Rights.^20^ As Dawson J notes in the Australian High Court, ‘the 1898 Constitutional Convention rejected a proposal to include an express guarantee of individual rights.… The framers preferred to place their faith in the democratic process for the protection of individual rights.’^21^ There are some protections embedded in the Constitution consistent with the perceived threats at the time of Federation; so, for instance, there are provisions relating to voting and direct representation,^22^ freedom from state-imposed religion,^23^ and trial by jury.^24^ More substantively, human rights protections, as they have evolved elsewhere, have been implied into the text and structure of the Constitution.^25^ These implications have included an implied freedom of political communication.^26^ Otherwise, the Constitution ‘with few exceptions … does not seek to establish personal liberty by placing restrictions upon the exercise of governmental power’,^27^ and the Australian High Court has generally declined to imply personal rights into the Constitution.^28^ There has been significant agitation for a Bill of Rights to be included in the Constitution; however, the requirements for amendment of the Constitution are elaborate,^29^ and few proposed amendments have been successful.^30^ Gelber notes that ‘there have been four attempts to introduce a Bill of Rights in either constitutional form through a referendum, or in statutory form by federal parliament—in 1973, 1983, 1985 and 1988. All failed in the context of a utilitarian political culture.’^31^

Australia has, however, ratified several international human rights conventions. Pursuant to High Court interpretations of s 51(xxix) of the Constitution (the external affairs power), conventions and treaties entered into by the executive^32^ can create a sufficient nexus to external affairs to enliven the jurisdiction of the federal Parliament to legislate with regard to the matters in the convention,^33^ with the caveat that the law must be ‘reasonably capable of being considered appropriate and adapted to implementing the treaty’.^34^

The implementing covenants of the Universal Declaration of Human Rights, the International Covenant on Civil and Political Rights (ICCPR),^35^ and the International Covenant on Economic, Social, and Cultural Rights (ICESCR)^36^ require ratifying states to legislate to protect enumerated freedoms, with defined exceptions. The ICCPR nominates powerful civil and political rights, including the right to liberty^37^ and freedom from arbitrary detention,^38^ freedom of movement and freedom to leave and enter their own country^39^ (with some exceptions such as public health orders),^40^ freedom of thought, conscience, and religion (including the freedom to worship in community, subject to lawful limitations necessary to protect public health),^41^ freedom of expression (subject to lawful restrictions necessary to protect public health).^42^ Notably, art 21 of the ICCPR requires state recognition of the right to peaceful assembly, which may be lawfully restricted conformably with national security, public safety or order, the ‘protection of public health or morals or the protection of the rights and freedoms of others’.^43^

In addition to the embedded limitations, art 4 of the ICCPR provides that state parties to the conventions may ‘take measures derogating from their obligations … to the extent strictly required by the exigencies of the situation.… [However,] [n]o derogation from articles 6, 7, 8 (paragraphs I and 2), 11, 15, 16 and 18 may be made under this provision.’^44^ The ICESCR enumerates rights such as the right to work,^45^ the right to the enjoyment of the ‘highest attainable standard of physical and mental health’ and ‘the prevention, treatment and control of epidemic, endemic, occupational and other diseases’,^46^ the right to education,^47^ and the right to take part in cultural life.^48^

Ratification does not affect domestic rights; however, ratification enlivens the external affairs power, which is part of the legislative grounding for a large number of federal enactments.^49^ At the federal level, the legislature has also passed the Human Rights (Parliamentary Scrutiny) Act 2011 (Cth). Developments in human rights, including as a result of the measures taken to combat COVID-19, are monitored by the Australian Human Rights Commission.^50^

Human rights protections have also been introduced at state and territory levels: the Human Rights Act 2004 (Australian Capital Territory), the Charter of Human Rights and Responsibilities 2006 (Vic),^51^ and the Human Rights Act 2019 (Queensland) contribute to the suite of protections,^52^ although of course in the event that they are inconsistent with federal legislation, the federal Act prevails. In Victoria, the Victorian Equal Opportunity and Human Rights Commission monitors and reviews human rights issues.^53^

The interaction between federal and state measures to combat COVID-19 is jurisdictionally important; whilst the strongest and potentially most entrenched human rights requirements are the obligations of the federal government, most COVID-19 measures, including lockdowns and travel restrictions, have been the actions of state governments.^54^ State public health legislation has been the primary driver of explicit constraints on the population, including constraints on movement.^55^ The federal response was considered to be one of ‘guidance and coordination’ as part of a ‘whole-of-government’ approach.^56^

## COVID and the potential for breach of human rights

It is clear, therefore, that fundamental rights are legitimately capable of proportionate curtailment in appropriate circumstances. Thus, public health emergencies, quarantine requirements, and certain requirements for the security of the population or the state are well-established exceptions to liberties such as freedom of movement and assembly. The World Health Organization (WHO) declared that COVID-19 was a public health emergency on 30 January 2020,^57^ and a pandemic on 11 March 2020,^58^ and it was then designated a notifiable disease in all Australian states and territories.^59^ Given the transmissibility of the virus and its potentially fatal consequences, it was appropriate for urgent measures to be taken. Concern for public health justifies a range of measures which may impact on some civil and political rights.

However, the extent and severity of measures, the mechanisms of enforcement, and the degree and effectiveness of oversight, remain valid concerns. The range of responses across Australian jurisdictions, the comparatively lengthy and burdensome constraints in the context of relatively low infection numbers, and the growing civil unrest aimed at restrictions on movement and employment, justify interrogation of government measures. The measures undertaken in Victoria, Australia, have included lockdowns of the city of Melbourne so frequent and so lengthy that it has reached the milestone of becoming the ‘world’s most locked down city’:^60^ ‘[t]he state capital on Monday chalked up 246 days living under stay-at-home orders across six lockdowns, surpassing the record set by the Argentinian capital of Buenos Aires.’^61^ The lockdown was to end by reference to a series of ‘roadmaps’ setting vaccination targets as conditions of re-opening.^62^ Lockdown conditions varied over time, but generally included bans on leaving the home except for specified reasons.^63^ Employment, other than for ‘authorised work’, was not one of those reasons.^64^ Stay at Home Directions (Restricted Areas) (No. 24), beginning 28 September 2021 and ending on 21 October 2021, included, for instance, a curfew from 9:00 p.m. to 5:00 a.m., and the requirement to wear masks both indoors and outdoors.^65^ Although restrictions included a period of exercise outside the home, the Directions prohibited arrangements to meet, organise, or intentionally attend a gathering for a common purpose.^66^

Curtailment of well-established human rights has been a concerning theme in reports from a number of jurisdictions.^67^ The WHO emphasised that, in their responses to COVID-19, ‘[a]ll countries must strike a fine balance between protecting health, minimizing economic and social disruption, and respecting human rights.’^68^ Regulatory controls during the COVID pandemic have included significant institution of border controls and travel bans, which have manifested in some cases in prevention of residents returning to their own country, mask mandates, curfews,^69^ stay-at-home orders,^70^ social distancing requirements,^71^ digital monitoring of citizen movement, prevention of gathering for worship,^72^ prevention of school attendance,^73^ and quarantine^74^ orders. Vaccination mandates^75^ have become a significant issue, particularly in the employment context. These regulations may apply to those not infected with the COVID virus, as well as to those who are. COVID regulatory measures have had cascading effects on other legal rights such as rights to property and contractual rights.^76^

At the same time, regulatory measures such as lockdowns have the potential to cause adverse health outcomes such as severe mental health issues and suicides,^77^ as well as medical issues arising from physical inactivity and reluctance to seek medical advice during a pandemic.^78^ Moreover, popular reactions to lockdowns can fuel ‘vaccine hesitancy’ or ‘anti-vaxxer’ messages in social media, anti-lockdown protest marches which can become COVID super-spreader events, and virulent anti-mask activity potentially resulting in violence.

Focussing on the Victorian jurisdiction, the legal validity of these measures derives from legislation, regulation, and public health directions. Procedures to manage the situation are set out in the Emergency Management Act 2013 (Vic), which creates the State Crisis and Resilience Council^79^ and subcommittees,^80^ Emergency Management Victoria,^81^ and the Emergency Management Commissioner,^82^ which is a statutory appointment.^83^ The control agency designated to deal with an emergency depends on whether it is a Class 1 or Class 2 emergency, and health emergencies are classified as Class 2 emergencies.^84^ The Department of Health and Human Services is the control agency using established protocols.^85^ The declaration of a state of emergency expanded the powers of the Chief Health Officer (CHO).^86^ The Public Health and Wellbeing Act 2008 (Vic) authorised officers to make public health orders or directions.^87^ Expansion of powers included the power to detain and restriction of movement. People could be prevented from entering an emergency area, which could include a large area such as a suburb, a municipality, or the state as a whole. Consequences of contravention in Victoria involved pecuniary penalties. Police powers were expanded to include the discretion to issue on-the-spot fines, along with the powers to arrest for breach of health directions, and police action was particularly marked by utilisation of ‘stop and search’ powers and the targeting of protesters.^88^ There were points of focus for those arguing that human rights had been, during this period, disproportionately curtailed. The lockdown of public housing blocks with little notice^89^ prompted concerns sharpened by the vulnerable communities therein and presented a parallel to the prolonged detention of refugees in Australia. The restriction of civil rights reached into the roots of legitimacy of democratic political culture because the rights to discuss, protest, and reach political agreement on the need for and extent of constraints were likewise constrained. This was exemplified by the handcuffing arrest of a pregnant woman in her own home, in front of her children, for posting support for protest action on a social media site.

### Locking down tower blocks

In July 2020 the Victorian Government made the decision to ‘lock down’ nine public housing blocks, or around 3,000 people, in Flemington and North Melbourne, preventing them from leaving their homes for five days.^90^ The legislative infrastructure authorising the lockdown necessitated a direction by the CHO (in this case the Deputy CHO with the authority to act on that day). Detention directions under the Public Health and Wellbeing Act 2008 (Vic) were made in relation to the premises on 04 July 2020. The hard lockdown occurred following the discovery that 23 residents of the tower blocks were amongst the 543 then-active cases across Victoria.^91^ Because of the crowded living conditions and shared community spaces the tower blocks were considered to be at high risk; however, the lockdown occurred with little notice: the Premier of Victoria announced that the lockdown would commence at 4:00 p.m. on the day of the announcement, whereas officials had understood that it would begin the following day so that residents could secure supplies.^92^ During the early stages of the lockdown, residents were denied access to fresh air and outdoor exercise. The Department of Health and Human Services (the Department) noted that human rights guidance on the requirement for fresh air and exercise was ‘developed in the context of imprisonment for criminal activity, not detention for infection control’,^93^ suggesting that the human rights of people detained for health reasons were different from—and less generous than—those detained for penal purposes. This ‘heavy-handed’^94^ action was subsequently criticised by human rights lawyers, community advocates, and the state Ombudsman.^95^

The context within which this action occurred is important: the tower blocks are a form of public housing in which resides a community which is already vulnerable in the sense that it does not have the financial resources available to the wider community. This vulnerability is exacerbated by an information deficit—many of the residents did not have English as a first language—and by the experience of some residents as immigrants from dictatorships, meaning that police action could be potentially traumatic.

In her investigation into the tower block lockdown, Ombudsman Deborah Glass noted that ‘[w]e may be tempted, during a crisis, to view human rights as expendable in the pursuit of saving human lives. This thinking can lead to dangerous territory.’^96^ The Ombudsman report appended the 44-page response of the Department to the draft report, in which, inter alia, the Department claimed that it was ‘*not wrong* for the Department to prioritise the protection of human life’,^97^ justifying the lockdown without notice, lack of access to exercise, timely and reasonable access to medication, and the failure to address human rights implications. In contrast to the dispassionate terms expected of a report, the Department appeared to be communicating exasperation that its critics did not view the situation as it did, as is evident by the following, non-exhaustive extracts:

the Department was responding to an emergency where human life was at stake, the aphorism that ‘perfection can be the enemy of the good’ is particularly relevant here. The Department’s response was *good*, even if not perfect.^98^

The Ombudsman’s draft findings would hold the Department to a standard that could not have been achieved while protecting lives to the very great extent that they could be protected, which was unashamedly the Department’s priority.^99^

The urgency with which the Department acted on 4 July 2020, is readily explained by [listed] self-evident matters. The Department then considered, and still considers, that *acting with immediate speed was absolutely necessary*.^100^

A review of the legislative context confirms that the Department’s primary focus on saving life and prioritising health above all else, was mandated by the PHW Act.^101^

Here we see the deployment of techniques of discursive legitimation in the framing of responses to criticism of the government measures. The Victorian State Government’s responses to the COVID-19 pandemic, in the context of clear challenges to accepted human rights, give rise to issues of legitimacy and legitimation. Even where it is possible to cite legal authority for the actions taken, there may be questions of validity on the basis of the proportionality of the responses. However, there are also clear questions arising from the use of techniques of legitimation of those actions. The framing of this response asserts the unassailable value of life above all other values, paradoxically creating the ‘bare life’ scenario considered below.^102^ Framing the narrative around the pandemic response in Victoria elevates certain salient facts, which simultaneously diminish or silence other views.^103^ Significant questions arise in the light of the Department response; prioritisation of health outcomes above all else is clearly disproportionate. Health outcomes would justify government interventions in all aspects of a person’s life, including compulsion to eat, drink, and exercise healthily. Inherently and unavoidably dangerous activities such as driving could be prohibited in the interests of personal safety. The community could, justifiably, be continuously detained for its own good, as well as the good of the citizenry generally. Moreover, the measures are based on authority which places the executive branch in the position of establishing the acceptable level of risk. The legislation creates a mechanism by which the executive can ‘recite itself into power’,^104^ and this power has few limits.

This event could also be considered from a racial justice perspective, prompting discussion of individual agency and the paternalistic policies that underscored white settlement of Australia. Government allocation and quantification of risk is central to the pandemic response, but the risks arising from the pandemic response itself fell disproportionately upon marginalised communities with insecure employment or/and low-quality housing. The high population of people from a non-English-speaking background, often newly arrived migrants without a firm hold in the Australian community, became the subjects of the exertion of control over all aspects of life and the body. Agamben argues that refugees are the ‘paradigmatic site—and victim—of … “governmentality”: the organised practices and techniques used to produce, care for and/or dominate individual subjects’, living, like the occupants of the tower blocks, in a ‘state of exception’.^105^ The exercise of the power to quarantine uses similar techniques as the processes of immigration and the processing of refugees. Just as refugees are reduced to ‘bare life’ whilst awaiting processing, the person in lockdown is stripped of all but the necessary food, shelter, and medication, and this is despite, in most cases, being a citizen of the Australian polity. Processes of lockdown, curfew, quarantine, COVID check-in, and the other governance techniques used during the pandemic produce the sort of ‘bare life’ some authors assert to be the outcomes of long-term refugee status.^106^ The exercise of power to exclude civil and human rights in such a marked way and for such a long period suggests that the use of detention as a solution to asylum-seeker claims is not juridically grounded in lack of citizenship, as is often asserted. Rather, the exercise of sovereign power on citizens and non-citizens alike finds legitimation through narrative alone—as long as the exercise of power meets acceptable levels of resistance, it can continue.

This prompts consideration of traditional processes of democratic legitimation. Ordinarily the democratic election of a government acts as proxy for the suite of legislative measures taken by that government whilst in power—the so-called ‘mandate’. In Australia, the implied freedom of political communication has been held to be a necessary incident of the polity created by the Constitution, and to prevent constraints on political speech. The prevention of political communication by restrictions on published speech and protest thus represents a more fundamental threat to legitimate governance. This will be considered by focussing not on the large protests to government COVID measures which characterised the later stages of the lockdowns, but on the arrest of a woman for the publication of a notice merely inviting protest.

### Policing political speech

The explicit silencing of certain manifestations of alternative views is represented by police action taken to suppress not only public assembly, but also the process of organising public dissent. On 02 September 2020, Zoe-Lee Buhler, a Ballarat mother of two, then pregnant with her third child, was arrested at home for inciting breach of public health laws through a social media post encouraging people to attend a ‘freedom day’ in Ballarat despite ‘stay at home’ orders.^107^ At the time, Ballarat was in Stage 3 lockdown, not the higher-constraint Stage 4 lockdown that applied in Melbourne at the time, and Buhler represented the projected protest as ‘a voice for those in stage four lockdowns’.^108^

She was charged under s 321G of the Crimes Act 1958 (Vic), which prohibits the pursuit of ‘a course of conduct which will involve the commission on an offence’.^109^ Her arrest was live-streamed (by her husband) and posted on Facebook. The video was extensively shared and viewed.^110^ The event manifested the twin themes of constraints on political speech and the use of police force in COVID-19 compliance measures. Lawyers recorded concerns about the arrest and the behaviour of police more generally in the enforcement of COVID measures. The president of the Victorian Bar, Wendy Harris QC, noted that ‘[t]he Victorian Bar is concerned that the enforcement response to Ms Buhler’s conduct is apparently at odds with other reported and more measured responses by authorities to organisers or promoters of similar protests planned or carried out in contravention of public health directives.’^111^ The Australian Human Rights Commission president, Rosalind Croucher, and Human Rights Watch spokesman Elaine Pearson, also expressed concern, along with a number of other legal advocates.^112^

There are several dimensions to the arrest which could attract genuine criticism. Police defended the actions of officers as ‘entirely reasonable’, ‘polite’, and ‘professional’ despite the ‘terrible optics’^113^ of the arrest of a pregnant woman in her own home, in her pyjamas, in front of her small children. Buhler herself asked why police did not merely telephone her and ask her to remove the post, describing herself as a law-abiding person who had a ‘bimbo moment’ and that she did not know what she was doing was illegal.^114^ The arrest could be seen as heavy-handed in this context, and disproportionate to the threat. The Victorian Bar’s reference to inconsistency in enforcement was a reference to the failure to take action against the organisers and participants in the ‘Black Lives Matter’ (BLM) protest which was held in June 2020 in Melbourne. The Victorian Equal Opportunity and Human Rights Commission ‘Explainer’ acknowledged that the right to peaceful assembly, freedom of association, and freedom of expression were set out in the Charter of Human Rights and Responsibilities but were subject to constraint by other laws and could be limited by necessary, justified, and proportionate measures.^115^ Referring to the apparent discrepancy between the treatment of BLM protests and other protests, the Commission said:

The Black Lives Matter protests were held in June 2020, before Victoria went into stage 4 lockdown, and protest organisers undertook to stage the protests in a manner that would comply with the CHO directions in place at the time. In June 2020, Victorians could leave home for any reason but the Stay Safe Directions required people to take reasonable steps to maintain a distance of 1.5 metres from other people and restricted gatherings outside to a maximum of 20 people. Black Lives Matter organisers encouraged protesters not to breach the restrictions and asked people to wear masks, bring hand sanitiser and remain 1.5 metres apart, ensuring distance between each group of 20 people.^116^

The Commission noted that fines were issued to three protest organisers for staging a protest that did not comply with the CHO’s directions, but otherwise did not address the restrictions on gathering which limited them to a maximum of 20 people.^117^

At the time of writing, Buhler’s prosecution has not been finalised, but her legal representative has indicated that her defence will include an argument that the public health directions at the time did not prohibit ‘peaceful, socially distanced protest’, whether Buhler had a ‘reasonable excuse under the Public Health and Wellbeing Act and whether she intended to breach the law or was just expressing a view’.^118^

As we have seen, the Constitution contains few express human rights protections, but some have been implied into its text and structure. Two of the relevant implied rights are the implied freedom of political communication^119^ and the separation of powers.^120^ Freedom of political communication, including protest action,^121^ entails an area of immunity from federal or state law on the basis that communication on matters of government and politics are ‘an indispensable incident of the system of representative government created by the Commonwealth Constitution’.^122^ However, the freedom may be curtailed by laws enacted for valid reason, as long as those laws are a proportionate response to a legitimate concern. A majority in the High Court in *McCloy v New South Wales*^123^ noted that the freedom ‘may be subject to legislative restrictions serving a legitimate purpose compatible with the system of representative government for which the Constitution provides, where the extent of the burden can be justified as suitable, necessary and adequate, having regard to the purpose of those restrictions’.^124^ The Victorian response to the COVID-19 pandemic contained explicit and wide-ranging constraints on political speech; most notably in the effect of ‘stay at home’ orders preventing massed political protest which were enforced against people joining rallies or demonstrations. Broadly speaking, the relevant legal question would be whether the constraints on political discussion introduced by the Public Health and Wellbeing Act 2008 (Vic) and regulations pursuant thereto were a proportionate response to the public health concerns created by the pandemic. The High Court in *Gerner v Victoria* considered that ‘limits on communication or movement which do not have a political character’ are not invalid constraints on political communication, and that because the plaintiff failed to plead that limits on freedom of movement burdened political communication, it had failed to prove its case.^125^ The proportionality of the extensive constraints on political action was not, therefore, fully considered. The question of constraints on political communication was raised in the early part of the lockdown, demonstrated by the arrest of Buhler, but became marked in the later months of the lockdowns when mass protests became more common.

Buhler’s arrest is emblematic of the sublimation of the political subject, with established civil rights, to the biological body governable as a result of the state of exception created by the pandemic. In the words of Agamben, refugees in long-term detention were in a state in which ‘the possibility of differentiating between our biological body and our political body—between what is incommunicable and mute and what is communicable and sayable—was taken from us forever’.^126^ What we see here, however, is that the artifice underlying the enumeration of rights as distinct from the person, or rather the person-as-body as distinct from the person-in-politic, creates opportunities for the systematic paring away of liberties. The state does not create the right to political speech; the process of political speech justifies the exertions of power by the state. However, when the exertion of power is framed in medical terms, and the decision as to what constitutes an ‘acceptable risk’ is determined by expert rather than democratic means, the technologies of governance mimic democratic processes and the true function of democratic speech is to justify measures that have already been taken. The narrative becomes strategic and is reliant on sophisticated narrative framing.

## Strategic narratives and discursive framing

Measures taken to respond to the COVID-19 pandemic have been marked by the utilisation of traditional media and vigorous social media responses. Spaces for discourse were created with the willing participation of the media. There have been few comparable opportunities for Australian state Premiers to carve out media coverage of up to an hour each day, outside catastrophic bushfire or flood emergencies, but unlike those transient emergencies the pandemic response enabled, in Victoria, 120 consecutive press conferences, ‘streamed live on television, social media accounts and news websites’.^127^ This became an expression of the urgency of the pandemic, and a projection of the seriousness of the government response. Victorian Premier Daniel Andrews became ‘the public face of the state’s response to the coronavirus pandemic not least through an uninterrupted series of … daily press conferences on his government’s actions’^128^ at 11:00 a.m. daily. The breadth of the narrative space created by this strategy mimicked a form of social construction of knowledge. The news conferences became both the authoritative source for legally significant constraints on the day-to-day movements of the population, but also the primary interface for construction of that knowledge. Premier Andrews, considered to have ‘strong communication skills and a nose for sniffing the political wind’,^129^ projected a ‘guardian’ persona in these conferences by speaking directly to the population: ‘[w]hen he addressed the media, he was talking directly to 60 per cent of the public who supported the health measures even if they were fatigued by lockdowns.’^130^

Allman suggests that Australian political leadership was influenced by the approach of Shane Fitzsimmons during the ‘Black Summer’ bushfires in 2019–2020: ‘The success of Fitzsimmons’ media strategy was immediately obvious. Daily press conferences soon became the go-to method for Australia’s leaders to communicate through later crisis periods of 2020.’^131^ Conversely, Christine Nixon, then Victorian Chief Commissioner of Police, was subjected to serious criticism when it was discovered that she had attended a hair appointment and met with her biographer during the Black Saturday bushfires.^132^ Prime Minister Scott Morrison’s vacation in Hawaii was cut short as a consequence of virulent criticism about his absence during bushfires in NSW in 2019, despite there being an acting Prime Minister and responses to bushfires being largely a state matter.^133^ The Premier of New South Wales, Gladys Berejiklian, was also subjected to criticism when she announced that she would be discontinuing the practice of personally conducting press briefings on the pandemic.^134^ In the current Australian political landscape the demand for the availability of a politician in a 24-hour news cycle has created a singular opportunity for politicians to construct the narrative, whilst conversely limiting the space and time available for journalistic interpretation of political statements.

Along with the monopoly on media space, the COVID press conferences enabled the engagement of manipulative techniques. Andrews sought to project an image of homely authenticity, even down to his clothing choices. Appearing to aim for the ‘fatherlike’, ‘decisive yet compassionate’^135^ approach associated with Fitzsimmons, he wore a black North Face jacket to project good news in press conferences, a jacket ‘more commonly associated with weekend bushwalkers or dads at soccer training (the jacket even spawned its own Facebook group)’.^136^ The jacket, like the public health messaging in the background of press conferences, conveyed meaning, and ‘[i]t’s unthinkable that Dan Andrews was unaware of the jacket’s symbolism, just as it’s unthinkable that he gave no thought as to what he’d wear to the press conferences, depending on the weight of the news he was about to deliver.’^137^

The televised updates were both material—containing case numbers (and fatalities) and updates on legal constraints—and discursive. The processes of narrative framing, buttressed by repetition and media amplification, were characterised by the phrases heightening the sense of risk. Repetition of zero-sum concepts became a frequent device. The phrase ‘saving lives’ was used repeatedly in daily press conferences. For instance, in reference to going to pubs and restaurants:

It will cost lives. It will cost lives.… If people simply behave as normal, if they don’t take this seriously, if they act selfishly, then people will die.… I don’t care what you call it, just do it. Because if you don’t, people will die. And you know who dies? The most vulnerable people in our Victorian community.… There’s nothing more serious than people dying, and certainly those who are the most vulnerable.… This is serious. This is absolutely serious and it needs to be taken seriously by every single Victorian. If it’s not, then people will die.^138^

Similar narrative processes of responsibilisation surround the use of the word ‘protect’: ‘[w]e’ve all got to own this at a very personal level because each of us, in the way we conduct ourselves, and the way we protect our family can, in turn, protect every Victorian family.’^139^ Invocation of the ‘family’ dynamic contributed to the manifestation of paternal authority which was also constructed by verbal and visual cues: Andrews displayed characteristic word choices signalling authority and discipline such as ‘make no mistake’, ‘be in no doubt about that’, ‘I want to make it very clear’,^140^ and ‘[t]his is appropriate. It’s painful, but it is absolutely appropriate’^141^—phrases which not only conveyed gentle rebuke but also unassailable correctness, as if appropriateness of government action is to be settled by the government itself. Phrases such as ‘surely that’s not too much to ask’^142^ deliberately evoke mild exasperation and simultaneously understate the very significant costs of lockdown (estimated at around $100 million a day in lost economic activity in Victoria, with debt levels almost tripling since 2019);^143^ costs which are not equally shared. Victoria, which arguably shared similar health risks from COVID, experienced a more significant impact on the economy because of the longer lockdowns.

Tones of exasperation and rebuke were not uncommon in Andrews’ press conferences. Where used in answer to journalists’ questions, they often followed a narrative independent of other interlocutors. An exasperated tone in response to journalists asking a benign question is an effective tactic to control the discursive space. The practice of carrying on an overarching narrative regardless of journalist questions renders the daily press conferences into a theatre for the viewers, not an open and constructive dialogue between journalists and government. For instance, the following exchanges occurred at a press conference early in the pandemic:^144^

[In response to a question about clear messaging in announcing the restrictions]: (Andrews without accepting the criticism): ‘Follow the advice and follow the rules. And what’s more, if you don’t, you’ve got every reason to believe that Victoria Police will catch you and you will be punished.’

[In response to a question about consistency between state and federal advice on school attendance]: (Andrews concludes): ‘The Government’s response will need to change from time to time. That’s the nature of these things. They move really fast and that’s frustrating. That’s challenging. But it’s nowhere near as challenging as people doing the wrong thing and then we have 10,000 people who can only survive if they’ve got a machine to help them breathe and we don’t have enough machines, nurses and doctors to get that job done.’

[In response to a question about the number of ICU beds available]: (Andrews, without giving the number of ICU beds, concludes): ‘But for those who are acting selfishly, it’s got to stop and it should.’

[In response to a question about the re-opening of schools): (Andrews, without giving a date, concludes): ‘Common sense says, and decency frankly, do the right thing.’

The overarching paternal approach appears to have been appropriately targeted; Murphy et al. note that surveys early in the pandemic demonstrated that ‘Australians cared less about health risks to them or to others and seemed more motivated to comply with COVID-19 restrictions out of a sense of duty to support authorities.’^145^

The press conferences were also supported by other buttressing mechanisms. In Victoria, the CHO also appeared at press conferences on a regular basis raising issues of the appropriate separation of powers. The role of the CHO during the pandemic has been subject to scrutiny in a number of jurisdictions:^146^ the CHO in Victoria is a public servant, and as part of the executive should be independent of the legislature. Public servants placed in this situation are at risk of undermining the public perception of their independence from government. The appearance of the CHO is also a useful form of social discipline alongside the other processes of medicalisation of daily interactions. The medical expertise of the CHO provides additional authority to the Premier and supports a population-level process of medical social control—medical surveillance.^147^ The medical gaze authorises both widespread digital monitoring (through check-in processes, vaccination screening, and COVID-tracing) but also privatises the processes and costs of surveillance by requiring businesses to check the status of entrants or face draconian fines.^148^ This subtle inversion of traditional citizen-state roles has the government mandating its own limits and the individual carrying out the state role of implementation of police measures.

Other narrative framing techniques associate positive values and periods of national struggle and emergency. Premier Andrews, for instance, described the virus situation as a ‘public health bushfire’^149^ and referred to people at the ‘frontline’ in the ‘fight’ against the virus. People were congratulated for their ‘sacrifice’.^150^ Concepts of ‘mateship’ were explicitly called upon.^151^ A language of singular struggle, of unanticipated crisis, is utilised with terminology such as ‘never before’^152^ and ‘unprecedented’:^153^ ‘[w]e have never done this before in any of our lifetimes, but it was appropriate and that’s why we’ve done it.’^154^ Bashford notes that a ‘language of defense of nation—resistance, protection, invasion, and immigration’^155^ has routinely been associated with Australian public health intervention. The geographic isolation which has enabled and influenced immigration policy has also created the capacity for ‘prophylactic quarantine’.^156^

The government strategy appears to have been largely successful in targeting ‘psychological similarities at both ends of the political spectrum (ie, left and right leaning), such as (a) their shared belief that the world is dangerous and (b) a common view that state control is needed to address those threats’.^157^ Bleakley notes that the Victorian response to the extensive lockdowns in fact demonstrates that the ‘general public largely remained supportive of restrictive lockdown measures throughout the crisis, indicating that it is possible to achieve compliance from the majority of the public in strictly enforced lockdowns, despite the intervention of small-but-enthusiastic sets of anti-lockdown activists.’^158^

Perhaps inevitably, however, as the lockdown measures were extended, and were joined by compulsory vaccination mandates, protests became more common. Non-compliance with lockdown restrictions in Victoria contributed to a surprising spike in cases in late 2021,^159^ even predating the Omicron variant, and despite the largely compliant attitude noted by Bleakley large protests against both lockdowns and compulsory vaccination requirements became a feature of Melbourne streets.^160^ Whilst these were often described as ‘anti-vaxxer’ protests^161^ in critical mainstream and social media, other concerns were manifested in the protests, including perceptions of police overreach, the economic costs on employer and small business associated with ensuring compliance with COVID status checking, employee shortages as a result of vaccination mandates, privacy concerns relating to the sharing of health information, and industry-specific concerns (for instance, relating to the two-week shutdown of the construction industry, which was the catalyst for several protest marches in Melbourne). The ‘tradie’ protests,^162^ along with the tower lockdowns, evidenced the disproportionate burden of lockdowns. For most of the lockdown period those who could work at home—typically white-collar and office workers and public servants—were largely uninterrupted in their labour and income. Other classes of ‘essential workers’ were obliged to work but both employee and employer were subject to heavy regulation. Tradespeople were constrained at points, but many could continue to work in regulated ways. Casualised workers in non-essential sectors were the most heavily affected by the lockdowns but had little capacity to effect political protest. Only when urban tradespeople were affected as a group did focussed protest occur.

A strategy in both government and popular responses to protest action has been the conflation of several concerns, some quite legitimate, to present all protest activity as an irrational and largely right-wing phenomenon. The use of narrative framing techniques colloquially known as ‘clickbait’ draw upon the most egregious statements or actions of protesters to attract readers to advertisers on news websites—focussing, perhaps, on references to ‘satanic ritual abuse’,^163^ conspiracies to implant trackers by vaccination, and protesters carrying ‘prop gallows’^164^ which were interpreted as direct threats to the physical safety of politicians.^165^ In the context of United States’ protests, Perrett argues that anti-lockdown protests became ‘magnets’ for right-wing extremism, and that although ‘protests over state-mandated social distancing and business closures weren’t started by extremist groups … white supremacists and those with similar ideologies have seized upon them’.^166^ The inability of organisers to control the narrative in a mass protest becomes a significant weakness, particularly in the face of the extensive and enclosed space afforded to the political class which had enabled it to construct the underlying ‘moral economy’.^167^ Where government is afforded a daily, extensive, and controlled right of reply, it becomes relatively simple to respond in a way that discredits the participants in protests as a whole.

Social media amplification of the government messaging has been particularly marked. The #IStandWithDan hashtag has been used to signal support of the government response, mainly by authentic accounts. It has also been a medium for virulent attacks against perceived opponents of the Premier.^168^ This is one of the ways in which social media exerts power over what may or may not be said. The paternal, comfortable dynamic set up by the pandemic press conferences is a theatre played to viewers, not to the press, and ‘[r]ather than wanting the media to closely question and pursue government, Twitter wants the media to back off and leave Dan alone.’^169^ The effect on criticism of administrative and political actions has been marked, with reports of academics feeling unable to engage in public discussion: Professor Catherine Bennet argues that ‘[t]he threat of a social media pile-on has discouraged some experts from contributing to the public debate.’^170^

A characteristic of the pandemic messaging has been the responsibilisation of the community as a whole. Characteristics of responsibilising messaging include the appeal to the collective good (‘we all have a part to play, it’s up to all of us to make this work’)^171^ and moral alignment with indisputable interests such as ‘vulnerable Victorians’ (‘to help protect those most at risk, new restrictions limiting access to aged care facilities are now in place’),^172^ health workers, or other essential workers. Businesses and employers are responsible for enforcement of vaccination, mask, check-in, cleaning, and density requirements. ‘Community members’ are encouraged to report breaches.^173^ The regulatory regime setting these protocols aligns the interests of the private enforcer with the state, as the penalties for breach by an organisation are more punitive than those for an individual. Responsibility is therefore devolved and detached from political decision-making. Dispersed governing mechanisms steer individual practices towards certain ends so that individuals and private organisations become regulators and willingly enforce COVID measures in order to continue to operate at all or to expedite the lifting of restrictions.

Conversely, management of the discursive space has been disrupted by the complex effects of multiple social media spaces, with their own processes of amplification, and some communities have chosen to use only selected forms of interaction.^174^ Social media criticisms of the government performance occurred (particularly aimed at perceived governmental overreach or mishandling of quarantine measures) and were linked to various Twitter hashtags (#DictatorDan, #ChairmanDan, and #DanLiedPeopleDied), matched by responses supportive of the Premier (#IStandWithDan).^175^ Bleakley argues that

the ‘Dictator Dan’ label that entered the Victorian cultural zeitgeist cannot be considered as an organic (or generalisable) public backlash to Andrews’s lockdown policy. Instead, the term (a) derived from a political opponent of Andrews, (b) was propagated on social media by a relatively small, yet vocal, minority of opponents, (c) many of these opponents posted from anonymous, unverifiable accounts and, perhaps most importantly, (d) the actual number of Tweets *supporting* Andrews was more than twice as high as those who perceived his actions as ‘dictatorial’.^176^

This suggests (at least in that social medium) that government narrative strategies to promote compliance were essentially successful. However, the government management of the message became more difficult as it transpired that government decision-making was the author of significant increases in COVID numbers. Failures in the mandated hotel quarantine programme were responsible for a number of deaths, ‘leading to 18,418 cases and 768 deaths, being 90% of all of Australia’s cases, and over 29,000 as of February 2021. Outbreaks at the Rydges and Stamford Plaza Hotels in May and June were traced back to these hotels through genomic sequencing.’^177^ A subsequent inquiry identified government failures in the programme,^178^ and the government’s own WorkSafe provisions resulted in charges against the Victorian Department of Health for breach of s 23(1) of the Occupational Health and Safety Act.^179^ Pressure also grew with the length and repetition of lockdown measures, and the ‘tradie’ protests, which occurred after the summary lockdown of construction industries in Melbourne.^180^ This presented a narrative challenge given that the Victorian Labor government is traditionally associated with the union movement: ‘[t]raditionally, labourism, as an ideology, represented a structure of concepts and associated practices directed towards the security of male manual wage earners and their families. These were the core of its constituency,’^181^ and one of the core values of the Andrews Labor government.^182^

## Never let a crisis go to waste

The foregoing account exposes the use of manipulative discursive techniques as a potential barrier to true discursive legitimacy. Whilst media management techniques are characteristic of modern government, the manipulative use of media, particularly social media, gives rise to serious legitimacy issues. Moreover, the use of these techniques over the longer-term risks declining efficacy, as can be seen by the growth of protest actions even in largely compliant Victoria. The process of backlash is a ‘key feature of how democratic societies function’,^183^ and

discourse can be both an instrument and an effect of power, but also a hindrance, a stumbling-block, a point of resistance and a starting point for an opposing strategy. Discourse transmits and produces power; it reinforces it, but also undermines and exposes it, renders it fragile and makes it possible to thwart it.^184^

Techniques of managing discourse and discursive framing align with processes of silencing the political person. Discourse analysis does not attempt to resolve issues of political legitimacy, but analysis of the use of language and the political implications of discourse does present salient questions for other theories of justice. The use of political communications for strategic purposes, using manipulative techniques, moves the outcome of political discussions further from the legitimated outcome. Much has been said about manipulation of social media discussions through social media algorithms^185^ and ‘fake news’,^186^ and there are many other ways in which individuals and large corporations can ‘game’ social media.^187^ Public relations itself could constitute an ‘engineering of consent’, a term originally used by Edward Bernays.^188^ Bernays himself considered public relations and propaganda to be interchangeable terms.^189^ In circumstances of clear limitations on established human rights, where those limitations have been noted with concern by impartial observers and government-instituted guardians of human rights, it is important to recognise the uses of power in discursive framing.

In the later stages of the pandemic the Victorian Parliament expanded legislative power to deal with pandemics, potentially at the expense of human rights and the rule of law.^190^ The legislation was introduced as the state of emergency declared in order to enable the suite of pandemic laws would expire on 16 December 2021. After its proposal the Bill prompted further waves of protest,^191^ and further processes of narrative framing to justify its breadth. It also prompted serious concern by legal commentators. Of the first iteration of the Bill, the president of the Victorian Bar, Christopher Blanden QC, noted concern about conferral of ‘draconian’ powers, ‘authorising virtually unlimited interference with the liberties of Victorian citizens [representing] the biggest challenge to the rule of law that this state has faced in decades’.^192^

A revised iteration was also subject to criticism by the Human Rights Law Centre on the grounds that the Premier, who under the bill replaces the CHO as the party able to declare a pandemic, does not need to demonstrate objectively reasonable grounds that there is a serious risk to public health.^193^ This iteration, whilst requiring extensions of the pandemic declaration every three months, allowed extensions for an indefinite period. Under this version there was no need for parliamentary approval, and the Bill did not explicitly require the Minister to properly consider and act compatibly with human rights. The Ombudsman, the Law Council, Liberty Victoria, and the Equal Opportunity and Human Rights Commission also raised concerns with the Bill,^194^ particularly in relation to the lack of a sunset clause, the capacity to detain a person without charge for an indefinite period, and the lack of a right of appeal to a court.

In other words, despite the opportunity afforded by months of focussed dialogue around the human rights consequences of COVID measures, the communication still appears to have been one-way. Only after negotiations with independent members of Parliament, forced by the surprising decision of a Labor parliamentarian to vote against the Bill,^195^ were some of the human rights concerns addressed. Thus, excessive fines for aggravated offences have been removed, an independent detention appeal panel is to be established, and a parliamentary review committee with a minority of government members is to have the power to recommend against pandemic orders. However, this will only be able to be achieved through a disallowance motion which would have to pass both houses of Parliament.^196^

## Conclusion

It is not an understatement to claim that COVID-19 responses have created the most significant and widespread challenges to Australian human, civil, and economic rights outside wartime. The relatively high levels of compliance are an interesting sidenote to the Australian population and may also be an effect of the successful application of discursive framing. The repercussions of the COVID-19 measures will continue to require monitoring; however, one of the insights of discourse analysis is the relative lack of effect of human rights monitoring during the course of the early pandemic. Human rights bodies exist at both state and federal levels in the Australian system, and trenchant criticism has been levelled at COVID measures by an independent Ombudsman, lawyers, and rights advocates. These criticisms appear to have had little impact on exertions of power.

No doubt the conversation about abrogation of rights will give rise to further agitation for a constitutional Bill of Rights, and it is appropriate periodically to revisit the debate about Australia’s lack of a constitutional Bill of Rights. However, the allowable scope of regulatory measures in pandemic circumstances is, arguably, not more difficult to draw in Australia, with no Bill of Rights, than in a country with a well-established Bill of Rights, since all rights are defined by reference to the risk to the state and its people. Fixed definitions in rights discourses acknowledge legitimate bases for abrogation of rights by insertion of legislative formulae such as ‘proportionate’ and ‘reasonable and appropriate’. These definitions are open to narrative framing.

This article demonstrated the existence of legislative and common law protections of human rights, and clear acceptance across the Australian community about the importance of human, civil, and political rights. However, and justifiably, these rights are subject to attenuation where overriding circumstances such as war and pandemic occur. The weakness in the human rights infrastructure is not in the existence of legislative or common law rights, but in the nature of language itself. Language around proportionality and reasonableness enables sophisticated narrative framing to justify continued constraints.

## Footnotes

1 Hanna E Huffstetler, Caitlin R Williams, and Benjamin M Meier, ‘Human Rights in Domestic Responses to the COVID-19 Pandemic: Preliminary Findings from a Media-Coverage Database to Track Human Rights Violations’ (2021) 9 *The Lancet Global Health* S16.

2 Sarah Repucci and Amy Slipowitz, *Democracy under Lockdown: The Impact of COVID-19 on the Global Struggle for Freedom* (Freedom House 2020) 1.

3 Public Health and Wellbeing Amendment (Pandemic Management) Act 2021 (Vic). See also ‘Summary of Victorian Bar’s Submission to the Department of Health and Expert Reference Group on the Public Health and Wellbeing (Pandemic Management) Bill 2021. https://www.vicbar.com.au/news-events/summary-victorian-bar%E2%80%99s-submission-department-health-and-expert-reference-group-public. Accessed 10 March 2022. See, further, media reactions including Bridget Rollason, ‘Victorian Premier Given Power to Declare Pandemics under Proposed Pandemic Laws’ (*ABC News*, 25 October 2021). https://www.abc.net.au/news/2021-10-25/victorian-premier-parliament-lockdown-pandemic-covid/100567664. Accessed 25 January 2022; Rachel Baxendale and Remy Varga, ‘Daniel Andrews Defends Proposed Laws Likened to “Stasi Powers” by Lawyers’ (*Australian*, 28 October 2021). https://www.theaustralian.com.au/nation/politics/lawyers-lash-daniel-andrews-stasi-covid19-pandemic-laws/news-story/cdcb1348832e2b3c7700856f13859c84. Accessed 07 March 2022.

4 Georges Enderle, ‘Re-learning the Human Rights Language: An Imperative for Our COVID-19 Era’ (2021) 2(1) *International Journal of Economics and Politics* 11, 13.

5 Simon Deakin and Gaofeng Meng, ‘The Governance of COVID-19: Anthropogenic Risk, Evolutionary Learning, and the Future of the Social State’ (2020) 49(4) *Industrial Law Journal* 539, 560.

6 Commonwealth of Australia Constitution Act 1900 (Imp) 63 & 64 Vict c 12, s 9 (‘the Constitution’).

7 Erin Handley, ‘Melbourne’s Coronavirus Curfew Has Been in Force for a Month. What Are the Pros and Cons?’ (*ABC News*, 04 September 2020). https://www.abc.net.au/news/2020-09-04/victoria-covid-19-curfew-pros-cons-health-response-policing/12626192. Accessed 07 March 2022.

8 See, for instance, Stay at Home Directions (Restricted Areas) (No. 15) 13 September 2020.

9 See, for instance, Stay Safe Directions (Metropolitan Melbourne) (No. 5) 23 October 2021; Victorian Border Crossing Order 2021 (No. 35) 21 October 2021.

10 Natalie Brown and Natalie Wolfe, ‘Coronovirus: Pregnant Victorian Woman Filmed Arrest’ (*News.com.au*, 03 September 2020). https://www.news.com.au/world/coronavirus/australia/coronavirus-pregnant-victorian-woman-filmed-arrest/news-story/cdd5dd3ed140416489c1925e4e6aef8d. Accessed 02 February 2022.

11 Sections 7, 24, and 128 of the Constitution combined have been interpreted as giving rise to an implied freedom of political communication. See also the discussion in Sect. 2 ‘The Australian Legal Landscape’.

12 *Australian Communist Party v Commonwealth* (1951) 83 CLR 1.

13 As was attempted in the Communist Party Dissolution Act 1950 (Cth), which was the subject of *Australian Communist Party v Commonwealth* (n 12), 206 (McTiernan J).

14 Kim Moloney and Susan Moloney, ‘Australian Quarantine Policy: From Centralization to Coordination with Mid-Pandemic COVID-19 Shifts’ (2020) 80(4) *Public Administration Review* 671, 672, citing Alison Bashford, ‘Quarantine and the Imagining of the Australian Nation’ (1998) 2(4) *Health: An Interdisciplinary Journal for the Social Study of Health, Illness and Medicine* 387, 388.

15 Ibid.

16 In *R v Kirby; Ex parte Boilermakers’ Society of Australia* (*Boilermakers’* case) (1956) 94 CLR 254, the High Court upheld a strict separation of the Commonwealth judicial power. In *Brandy v Human Rights and Equal Opportunity Commission* (1995) 183 CLR 245, the High Court held that the judicial power of the Commonwealth can only be exercised by courts established under Ch III of the Constitution. In *Chu Kheng Lim v Minister for Immigration, Local Government and Ethnic Affairs* (1992) 176 CLR 1, the High Court held that Parliament may not impermissibly intrude into the exercise of judicial power.

17 See generally Anne Twomey and Glenn Withers, *Federalist Paper 1: Australia’s Federal Future* (Report for the Council for the Australian Federation, 2007).

18 *University of Wollongong v Metwally* (1984) 158 CLR 447.

19 Section 71 of the Constitution provides that the judicial power of the Commonwealth is vested in the High Court, and s 75 establishes the original jurisdiction of the Court. Section 76 confers additional original jurisdiction to include matters rising under the Constitution or involving its interpretation.

20 See generally Katharine Gelber, ‘The Universal Declaration of Human Rights at 70: Protection of Human Rights in Australia’ (2019) 73(4) *Australian Journal of International Affairs* 313.

21 *Kruger v Commonwealth* (1997) 190 CLR 1, 61 [Dawson J].

22 Effectively protected by ss 7, 24, and 41 of the Constitution, supplemented by ss 1, 8, 13, 25, 28, and 30. See also *Lange v Australian Broadcasting Corporation* (1997) 189 CLR 520.

23 Section 116 provides that ‘[t]he Commonwealth shall not make any law for establishing any religion, or for imposing any religious observance, or for prohibiting the free exercise of any religion, and no religious test shall be required as a qualification for any office or public trust under the Commonwealth.’ See further *Adelaide Company of Jehovah’s Witnesses v Commonwealth* (1943) 67 CLR 116; *Church of the New Faith v Commissioner of Pay-Roll Tax (Vic)* (*Scientology* case) (1983) 154 CLR 120; *Attorney-General (Vic) (Ex rel Black) v Commonwealth* (the *DOGS* case) (1981) 146 CLR 559.

24 Section 80 of the Constitution states: ‘The trial on indictment of any offence against any law of the Commonwealth shall be by jury, and every such trial shall be held in the State where the offence was committed, and if the offence was not committed within any State the trial shall be held at such place or places as the Parliament prescribes.’ See also *Brown v The Queen* (1986) 160 CLR 171. However, see also Amelia Simpson and Mary Wood, ‘“A Puny Thing Indeed”—*Cheng v The Queen* and the Constitutional Right to Trial by Jury’ [2001] *Federal Law Review* 4.

25 See generally Dan Meagher, ‘The Judicial Evolution (or Counter-revolution) of Fundamental Rights Protection in Australia’ (2017) 42(1) *Alternative Law Journal* 9.

26 As noted earlier in (n 11).

27 *Kruger v Commonwealth* (n 21), 61 [Dawson J].

28 Given the restrictions on travel between states prompted by pandemic concerns, it is worth noting that s 92 of the Australian Constitution restricts the passage of laws interfering with movement of goods and persons across state borders: see Shipra Chordia, ‘Border Closures, COVID-19 and s 92 of the Constitution—What Role for Proportionality (If Any)?’ (*Australian Public Law*, 05 June 2020). https://www.auspublaw.org/blog/2020/06/border-closures-covid-19-and-s-92-of-the-constitution?rq=chordia. Accessed 29 April 2022.

29 Section 128 of the Constitution requires manner and form requirements for amendment, entailing approval by electors in a referendum both nationally and in a majority of states.

30 Mohammad Ibrahim, ‘Constitutional Change: Towards Better Human Rights Protection in Australia’ (2019) 5 *Constitutional Review* 248.

31 Gelber, ‘The Universal Declaration of Human Rights at 70’ (n 20) 314.

32 The Federal Executive has the capacity to enter into conventions and treaties under s 61 of the Constitution.

33 *Commonwealth v Tasmania* (*Tasmanian Dam* case) (1983) 158 CLR 1; *Richardson v Forestry Commission* (1988) 164 CLR 261; *Queensland v Commonwealth* (*Tropical Rainforests* case) (1989) 167 CLR 232; *Victoria v Commonwealth* (*Industrial Relations* case) (1996) 187 CLR 416.

34 *Victoria v Commonwealth* (*Industrial Relations* case) (n 33) [34].

35 International Covenant on Civil and Political Rights (ICCPR). https://www.ohchr.org/en/professionalinterest/pages/ccpr.aspx. Accessed 30 November 2021.

36 International Covenant on Economic, Social and Cultural Rights (ICESCR). https://www.ohchr.org/en/professionalinterest/pages/cescr.aspx. Accessed 31 January 2022.

37 ICCPR (n 35) art 12.

38 Ibid. art 9.

39 Ibid. art 12(2).

40 Ibid. art 12(3).

41 Ibid. art 18.

42 Ibid. art 19.

43 Ibid. art 21. See also ICCPR art 22 relating to freedom of association.

44 Ibid. art 4(2).

45 ICESCR (n 36) art 6.

46 Ibid. art 12.

47 Ibid. art 13.

48 Ibid. art 15.

49 Explicit human rights legislation at federal level includes the Age Discrimination Act 2004 (Cth), the Disability Discrimination Act 1992 (Cth), the Racial Discrimination Act 1975 (Cth), the Sex Discrimination Act 1984 (Cth), and the Australian Human Rights Commission Act 1986 (Cth). Ratification also grounds a number of other enactments which incidentally protect human rights, including legislation protecting working conditions and other workplace rights.

50 Australian Human Rights Commission, ‘What Is the Commission’s View on Limiting Human Rights during COVID 19?’. https://humanrights.gov.au/about/COVID19-and-human-rights/what-commissions-view-limiting-human-rights-during-COVID-19. Accessed 30 November 2021.

51 See generally Russell Solomon, ‘Reviewing Victoria’s Charter of Rights and the Limits to Our Democracy’ (2017) 42(3) *Alternative Law Journal* 195.

52 See also, in Victoria, the Equal Opportunity Act 2010 (Vic), the Racial and Religious Tolerance Act 2001 (Vic), and the Change or Suppression (Conversion) Practices Prohibition Act 2021 (Vic).

53 Charter of Human Rights and Responsibilities Act 2006 (Vic) s 41.

54 Australian Human Rights Commission, ‘COVID19 and Human Rights’. https://humanrights.gov.au/about/COVID19-and-human-rights. Accessed 30 November 2021.

55 Public Health Act 1997 (ACT), Public Health Act 2020 (NSW), Public and Environmental Health Act 2011 (NT), Public Health Act 2005 (Qld), South Australian Public Health Act 2011 (SA), Public Health Act 1997 (Tas), Public Health and Wellbeing Act 2008 (Vic), Public Health Act 2016 (WA). See for instance Restricted Activity Directions (Victoria) (No. 7) made pursuant to the Public Health and Wellbeing Act 2008 (Vic) s 200, which directed a restricted activity period commencing 12 February 2021 that prevented attendance at work unless exempt, and the Stay Safe Directions (Regional Victoria) (No. 15) Public Health and Wellbeing Act 2008 (Vic) s 200, which prevented residents of regional Victoria from travelling to metropolitan areas.

56 Australian Government Department of Health, *Australian Health Sector Emergency Response Plan for Novel Coronavirus* (Commonwealth of Australia 2020) 5.

57 Statement on the Second Meeting of the International Health Regulations (2005) Emergency Committee regarding the Outbreak of Novel Coronavirus (2019-nCoV) (30 January 2020). https://www.who.int/news/item/30-01-2020-statement-on-the-second-meeting-of-the-international-health-regulations-(2005)-emergency-committee-regarding-the-outbreak-of-novel-coronavirus-(2019-ncov). Accessed 07 March 2022.

58 World Health Organization, ‘WHO Director-General’s Opening Remarks at the Media Briefing on COVID-19’ (11 March 2020). https://www.who.int/director-general/speeches/detail/who-director-general-s-opening-remarks-at-the-media-briefing-on-COVID-19—11-march-2020. Accessed 24 November 2021.

59 Public Health (Novel Coronavirus—Temporary Notifiable Condition) Declaration 2020 (ACT) made under the Public Health Act 1997 s 101(a) and (b), effective until 03 August 2020; Public Health Amendment (Scheduled Medical Conditions and Notifiable Diseases) Order 2020 (NSW) made under the Public Health Act 2010 ss 51(2) and 81; Declaration and Notification of Notifiable Disease Novel Coronavirus (2019-nCoV) Infection made under s 6 of the Notifiable Diseases Act 1981 (NT); Public Health (Coronavirus [2019-nCoV]) Amendment Regulation 2020 under the Public Health Act 2005 (Qld); Declaration under s 63(2) of the Public Health Act 2011 (SA); Order of the Director of Public Health, acting pursuant to s 40 of the Public Health Act 1997 (Tas); Public Health and Wellbeing Amendment (Coronavirus) Regulations 2020 No. 4 (Vic) made under the Public Health and Wellbeing Act 2008; Public Health (Notifiable Infectious Diseases) Order 2020 (WA) made under Public Health Act 2016 s 90(2) in effect for six months from the date it was made.

60 This phrase originates from a statement by the Federal Treasurer Josh Frydenberg: ‘Josh Frydenberg Says Melbourne Is the World’s Most Locked Down City. Is That Correct?’ (*ABC News*, 25 October 2021). https://www.abc.net.au/news/2021-10-25/fact-check-is-melbourne-most-locked-down-city/100560172. Accessed 07 March 2022. Although the ABC fact check found the claim ‘not clear cut’, it has been reproduced in many news articles. See, for instance, ‘Melbourne Reopens as World’s Most Locked-Down City Eases Limits’ (*Al Jazeera*, 22 October 2021). https://www.aljazeera.com/news/2021/10/22/update-2-melbourne-reopens-as-worlds-most-locked-down-city-eases-pandemic-restrictions. Accessed 07 March 2022.

61 ‘Victoria Launches Indigenous COVID-19 Vaccine Push as it Records 1377 New Cases’ (*SBS News*, 4 October 2021). https://www.sbs.com.au/news/victoria-launches-indigenous-covid-19-vaccine-push-as-it-records-1-377-new-cases/ac4ed681-8af8-40cf-ad07-4aba28d39afa. Accessed 29 April 2022; Judd Boaz, ‘Melbourne Passes Buenos Aires’ World Record for Time Spent in COVID-19 Lockdown’ (*ABC News*, 03 October 2021). https://www.abc.net.au/news/2021-10-03/melbourne-longest-lockdown/100510710. Accessed 02 February 2022.

62 Premier of Victoria The Hon Daniel Andrews, ‘Victoria’s Roadmap: Delivering the National Plan’ (19 September 2021). https://www.premier.vic.gov.au/victorias-roadmap-delivering-national-plan. Accessed 07 March 2022.

63 Victorian Government, ‘COVIDSafe Settings’. https://www.coronavirus.vic.gov.au/coronavirus-COVIDsafe-settings. Accessed 04 October 2021.

64 Victorian Government, ‘How We Live: Information for Victorians’. https://www.coronavirus.vic.gov.au/authorised-provider-and-authorised-worker-list. Accessed 30 November 2021.

65 Section 1(4) of the Directions from Acting Chief Health Officer in accordance with emergency powers arising from declared state of emergency, Stay at Home Directions (Restricted Areas) (No. 24) 28 September 2021.

66 Ibid.

67 See, for instance, V Ibezim-Ohaeri and Z Ibeh, ‘The Civic Space in Nigeria: Before and Beyond COVID-19’ (Spaces for Change, 2020); Eric Mykhalovskiy et al., ‘Human Rights, Public Health and COVID-19 in Canada’ (2020) 111(6) *Canadian Journal of Public Health* 975, 979.

68 World Health Organization, ‘WHO Director-General’s Opening Remarks’ (n 58).

69 The validity of a curfew imposed by the Stay at Home Directions (Restricted Areas) (No. 15) was considered by the Victorian Supreme Court in *Loielo v Giles* (2020) VSC 722.

70 See, for instance, Stay at Home Directions (n 8).

71 Ibid. cl 9.

72 See, for instance, Stay at Home Directions (n 8).

73 Ibid.

74 See, for instance, Diagnosed Persons and Close Contacts Directions (No. 31) 23 October 2021.

75 See, for instance, COVID-19 Mandatory Vaccination (Workers) Directions 7 October 2021.

76 See generally Janice Lee, ‘Note on COVID-19 and the Police Powers Doctrine: Assessing the Allowable Scope of Regulatory Measures during a Pandemic’ (2020) 13 *Contemporary Asia Arbitration Journal* 229; Joseph J Amon and Margaret Wurth, ‘A Virtual Roundtable on COVID-19 and Human Rights with Human Rights Watch Researchers’ (2020) 22(1) *Health and Human Rights Journal* 399; Alessandra Spadaro, ‘COVID-19: Testing the Limits of Human Rights’ (2020) 11(2) *European Journal of Risk Regulation* 317.

77 Balasankar Ganesan et al., ‘Impact of Coronavirus Disease 2019 (COVID-19) Outbreak Quarantine, Isolation, and Lockdown Policies on Mental Health and Suicide’ (2021) 12 *Frontiers in Psychiatry* 565190.

78 Liam Mendes, ‘Skin Cancer Warning for Locked Down States’ (*Australian*, 29 November 2021). https://www.theaustralian.com.au/nation/skin-cancer-warning-for-locked-down-states/news-story/295b508552813afff7d5915ef21f6709. Accessed 07 March 2021.

79 Emergency Management Act 2013 (Vic) s 6.

80 Ibid. s 10.

81 Ibid. s 14.

82 Ibid. s 24.

83 Ibid. s 25.

84 Ibid. s 3.

85 Emergency Management Victoria, *State Health Emergency Response Plan* (4th edn, Victorian Government 2017) 21–22; Emergency Management Victoria, *Victorian Action Plan for Influenza Pandemic* (Victorian Government 2015).

86 Public Health and Wellbeing Act 2008 (Vic) ss 198–203. See Yara Murray-Atfield, ‘Victoria Has Declared a State of Emergency because of Coronavirus COVID-19. Here’s What That Means’ (*ABC News*, 16 March 2020). https://www.abc.net.au/news/2020-03-16/victoria-coronavirus-state-of-emergency-powers-explained/12059194. Accessed 02 February 2022.

87 Victorian COVID-19 Public Health Directions are publicly available online, including through http://classic.austlii.edu.au/au/legis/vic/covid-19_dir/. Accessed 29 April 2022. See generally Holly McLean and Ben Huf, ‘Emergency Powers, Public Health and COVID-19’, Research Paper No. 2 (Parliament of Victoria 2020).

88 Louise Boon-Kuo et al., ‘Policing Biosecurity: Police Enforcement of Special Measures in New South Wales and Victoria during the COVID-19 Pandemic’ (2021) 33(1) *Current Issues in Criminal Justice* 76, 80.

89 Philip Russo, ‘Nine Melbourne Tower Blocks Put into “Hard Lockdown”—What Does It Mean, and Will It Work?’ (*Conversation*, 05 July 2020). https://theconversation.com/nine-melbourne-tower-blocks-put-into-hard-lockdown-what-does-it-mean-and-will-it-work-142033. Accessed 02 February 2022; Rachel Baxendale, ‘Melbourne Tower Lockdowns Could Have “Horrific Consequences”, Victorian Government Told’ (*Australian*, 05 July 2020). https://www.theaustralian.com.au/nation/melbourne-tower-lockdowns-could-have-horrific-consequences-victorian-government-told/news-story/3755c1c3e6bc59ff1c3a75f3944d8a4a. Accessed 07 March 2022.

90 Ibid.

91 Russo, ‘Nine Melbourne Tower Blocks Put into “Hard Lockdown”’ (n 89).

92 ‘Lockdown of Victoria’s Public Housing Towers during COVID Crisis Breached Human Rights, Ombudsman Finds’ (*ABC News*, 17 December 2020). https://www.abc.net.au/news/2020-12-17/lockdown-public-housing-towers-breached-human-rights-ombudsman/12991162. Accessed 02 February 2022.

93 Victorian Ombudsman, *Investigation into the Detention and Treatment of Public Housing Residents arising from a COVID-19 ‘Hard Lockdown’ in July 2020* (17 December 2020) (henceforth *Victorian Ombudsman*) 101 https://www.ombudsman.vic.gov.au/our-impact/investigation-reports/investigation-into-the-detention-and-treatment-of-public-housing-residents-arising-from-a-covid-19-hard-lockdown-in-july-2020/. Accessed 29 April 2022.

94 Rollason, ‘Victorian Premier Given Power to Declare Pandemics’ (n 3).

95 *Victorian Ombudsman* (n 93).

96 Ibid. 5.

97 Department of Health and Human Services, ‘Response to the Ombudsman’s Draft Report’, Appendix A in *Victorian Ombudsman* (n 93) 184.

98 Ibid. 184.

99 Ibid. 184-5.

100 Ibid. 185 (emphasis in original).

101 Ibid. 192.

102 Patricia Owens, ‘Reclaiming “Bare Life”? Against Agamben on Refugees’ (2009) 23(4) *International Relations* 568.

103 Arianna Bove, ‘Politics without Romance? The Pursuit of Consent in Democracy’ (2020) 46(3) *History of European Ideas* 325, 330. See also Edward S Herman and Noam Chomsky, *Manufacturing Consent: The Political Economy of the Mass Media* (Random House 2010).

104 *Australian Communist Party v Commonwealth* (n 12), 206 (McTiernan J).

105 Owens, ‘Reclaiming “Bare Life”?’ (n 102).

106 See, for instance, Jenny Edkins and Véronique Pin-Fat, ‘Through the Wire: Relations of Power and Relations of Violence’ (2005) 34(1) *Millennium: Journal of International Studies* 1; Fiona Jenkins, ‘Bare Life: Asylum-Seekers, Australian Politics and Agamben’s Critique of Violence’ (2004) 10(1) *Australian Journal of Human Rights* 79.

107 Kelsey Rettino, ‘Ballarat Woman Live-Streams Arrest for Allegedly Inciting Coronavirus Lockdown Protest on Facebook’ (*ABC News*, 02 September 2020). https://www.abc.net.au/news/2020-09-02/woman-charged-ballarat-covid-protest-live-stream/12623118?utm_campaign=abc_news_web&utm_content=link&utm_medium=content_shared&utm_source=abc_news_web. Accessed 12 March 2022.

108 Michael McGowan, ‘Pregnant Woman Arrested in Ballarat for Creating Anti-lockdown Protest Event on Facebook’ (*Guardian*, 02 September 2020). https://www.theguardian.com/australia-news/2020/sep/02/three-men-arrested-ahead-of-planned-anti-lockdown-protest-in-victoria. Accessed 12 March 2022.

109 Naaman Zhou, ‘Victorian Bar Criticises Arrest of Pregnant Woman for Facebook Lockdown Protest Post as “Disproportionate”’ (*Guardian*, 03 September 2020). https://www.theguardian.com/australia-news/2020/sep/03/victoria-police-arrested-pregnant-woman-facebook-post-zoe-buhler-australia-warn-lockdown-protesters. Accessed 12 March 2022.

110 See for instance BBC (UK) coverage, ‘COVID: Australian Anti-lockdown Suspect’s Arrest Draws Controversy’ (*BBC News*, 03 September 2020). https://www.bbc.com/news/world-australia-54007824. Accessed 02 February 2022; Imogen Braddick, ‘Pregnant Mum Arrested and Charged for Allegedly Advocating against Coronavirus Lockdown in Australia’ (*Evening Standard*, 03 September 2020). https://www.standard.co.uk/news/world/australia-mum-arrested-lockdown-protest-a4539261.html. Accessed 02 February 2022; Natalie O’Neill, ‘Pregnant Australian Mom Arrested for Facebook Post Planning Lockdown Protest’ (*New York Post*, 02 September 2020). https://nypost.com/2020/09/02/pregnant-mom-arrested-for-facebook-post-planning-lockdown-protest/. Accessed 02 February 2022; Brown and Wolfe, ‘Coronovirus: Pregnant Victorian Woman Filmed Arrest’ (n 10).

111 Victorian Bar, Media Release, 03 September 2020. https://www.vicbar.com.au/news-events/media-release-%E2%80%93-3-september-2020. Accessed 30 November 2021.

112 See Binoy Kampmark, ‘Human Rights and Pandemic Lockdowns’ (2020) 30(18) *Eureka Street*.

113 Simone Fox Koob, ‘Police Stand by Arrest of Pregnant Mum but Handcuffing Stokes Debate’ (*Age*, 03 September 2020). https://www.theage.com.au/national/victoria/pregnant-ballarat-mum-blames-bimbo-moment-for-lockdown-protest-20200903-p55rz1.html. Accessed 05 February 2022.

114 Ibid.

115 Victorian Equal Opportunity and Human Rights Commission, *Explainer: Protests during COVID-19*. https://www.humanrights.vic.gov.au/resources/explainer-protests-during-COVID-19/. Accessed 12 March 2022.

116 Ibid.

117 Ibid.

118 Caroline Schelle, ‘Pregnant Ballarat Mum Zoe Buhler to Argue Police Search Wasn’t Legal’ (*Australian*, 27 March 2021). https://www.theaustralian.com.au/breaking-news/pregnant-ballarat-mum-zoe-buhler-to-argue-police-search-wasnt-legal/news-story/0ff35793350adc2a8b319a5a48605d09. Accessed 05 February 2022.

119 See (n 11) and *Lange v Australian Broadcasting Corporation* (n 22).

120 See (n 16).

121 *Levy v Victoria* (1997) 189 CLR 579; *Brown v Tasmania* (2017) 261 CLR 328.

122 *Lange v Australian Broadcasting Corporation* (n 22).

123 *McCloy v New South Wales* (2015) 257 CLR 178.

124 Ibid. (French CJ, Kiefel, Bell, and Keane JJ).

125 *Gerner v Victoria* [2020] HCA 48; 95 ALJR 107.

126 Giorgio Agamben, *Homo Sacer: Sovereign Power and Bare Life* (Daniel Heller-Roazen tr, Stanford University Press 1998) 188.

127 ‘Daniel Andrews Is Finally Taking a Day Off. Is This a Sign Things Are Looking Up for Victoria?’ (*ABC News*, 30 October 2020). https://www.abc.net.au/news/2020-10-30/daniel-andrews-takes-day-off-after-120-covid-media-briefings/12831460. Accessed 12 March 2022.

128 Timothy Graham et al., ‘#IStandWithDan versus #DictatorDan: The Polarised Dynamics of Twitter Discussions about Victoria’s COVID-19 Restrictions’ (2021) 179(1) *Media International Australia* 127, 128.

129 Annika Smethurst, ‘Dan the Untouchable: Why Scandal Fails to Trouble the Premier’ (*Age*, 10 October 2021). https://www.theage.com.au/politics/victoria/dan-the-untouchable-why-scandal-fails-to-trouble-the-premier-20211008-p58yfz.html. Accessed 05 February 2022.

130 Ibid., citing Labor’s former Assistant State Secretary turned pollster Kosmos Samaras.

131 Kate Allman, ‘Authentic Leadership: Shane Fitzsimmons’ (2021) 78 *Law Society of NSW Journal* 30, 33.

132 ‘Nixon at Hairdresser on Black Saturday’ (*Sydney Morning Herald*, 16 April 2010). https://www.smh.com.au/national/nixon-at-hairdresser-on-black-saturday-20100416-sik9.html. Accessed 05 February 2022.

133 The scope of Commonwealth powers is limited to those specified in the Constitution (with the implied nationhood power potentially increasing their scope). State powers are plenary powers, subject to the Constitution. Bushfires are not mentioned in the Constitution. See generally Royal Commission into National Natural Disaster Arrangements, ‘Background Paper: Constitutional Issues and National Natural Disaster Arrangements’ (22 May 2020). https://naturaldisaster.royalcommission.gov.au/system/files/2020-05/Background%20paper%20-%20Constitutional%20issues%20and%20national%20natural%20disaster%20arrangements.pdf. Accessed 12 March 2022.

134 ‘NSW COVID-19 Update: Berejiklian to Stop Daily Press Briefing despite Record High 1542 Cases—Video’ (*Guardian*, 10 September 2021). https://www.theguardian.com/australia-news/video/2021/sep/10/nsw-covid-19-update-berejiklian-to-stop-daily-press-briefing-despite-record-high-1542-cases-video. Accessed 05 February 2022.

135 Allman, ‘Authentic Leadership’ (n 131) 33.

136 Melissa Singer, ‘When Dan Finally Had Good News, He Had to Wear the Right Clothes’ (*Sydney Morning Herald*, 26 October 2020). https://www.smh.com.au/lifestyle/fashion/when-dan-finally-had-good-news-he-had-to-wear-the-right-clothes-20201026-p568qk.html. Accessed 05 February 2022.

137 Ibid.

138 See for instance, Daniel Andrews Press Conference (23 March 2020), recorded by 10 News First Melbourne. https://www.facebook.com/10NewsMelb/videos/1129253020742617. Accessed 12 March 2022.

139 Daniel Andrews Press Conference (07 July 2020), recorded by 10 News First Melbourne. https://www.facebook.com/watch/live/?ref=watch_permalink&v=196568245106672. Accessed 12 March 2022.

140 Daniel Andrews Press Conference (19 September 2021), recorded by 7 News Melbourne. https://www.facebook.com/watch/live/?ref=watch_permalink&v=1296498900810011. Accessed 12 March 2022.

141 Daniel Andrews Press Conference (23 March 2020) (n 138).

142 Ibid.

143 Patrick Commins, ‘Victoria’s Post-COVID Fiscal Position Will Be Worst in the Nation, S&P Warns’ (*Australian*, 29 November 2021). https://www.theaustralian.com.au/business/victorias-postcovid-fiscal-position-will-be-worst-in-the-nation-sp-says/news-story/18201b37ff41975ca71d59005bd2472e. Accessed 07 March 2022; see also Patrick Abraham et al., ‘Yes, Lockdowns Are Costly. But the Alternatives Are Worse’ (*Conversation*, 01 July 2021). https://theconversation.com/yes-lockdowns-are-costly-but-the-alternatives-are-worse-163572. Accessed 05 February 2022.

144 All exchanges occurred at the Daniel Andrews Press Conference (23 March 2020) (n 138).

145 Kristina Murphy et al., ‘Why People Comply with COVID-19 Social Distancing Restrictions: Self-Interest or Duty?’ (2020) 53(4) *Australian and New Zealand Journal of Criminology* 477, 489.

146 Margaret MacAulay et al., ‘Under the Spotlight: Understanding the Role of the Chief Medical Officer in a Pandemic’ (2021) 76(1) *Journal of Epidemiology and Community Health* 1.

147 Peter Conrad, ‘Medicalization and Social Control’ (1992) 18(1) *Annual Review of Sociology* 209, 216.

148 Open Premises Directions (No. 7) commenced at 6:00 p.m. on 25 November 2021.

149 Daniel Andrews Press Conference (23 March 2020) (n 138).

150 Daniel Andrews Press Conference (04 July 2020), recorded by 10 News First Melbourne. https://www.facebook.com/watch/?v=271287307540012. Accessed 27 April 2022.

151 Daniel Andrews Press Conference (23 March 2020) (n 138).

152 Ibid.

153 Ibid.

154 Ibid.

155 Bashford, ‘Quarantine and the Imagining of the Australian Nation’ (n 14) 388.

156 Ibid. 393, citing the ‘Report on Quarantine’, NSW Legislative Assembly, Votes & Proceedings, 1883: 5.

157 Paul Bleakly, ‘The Fight to Remain Compliant: Public Sentiment, Pandemic and Policing the Second 2020 Victorian Lockdown’ (2021) 1(1) *Journal of Contemporary Crime, Harm, and Ethics* 23, 32.

158 Ibid. (Abstract).

159 John Ferguson, ‘Rise in Defiance of Victorian Rules, for Sanity’s Sake’ (*Australian*, 02 October 2021). https://www.theaustralian.com.au/nation/politics/rise-in-defiance-of-victorian-rules-for-sanitys-sake/news-story/d1818316b8250175c88cd78b69c7bcb6. Accessed 07 March 2022.

160 Alex Druce and Frances Vinall, ‘Police Surround Shrine of Remembrance as Anti-lockdown Protesters Gather’ (*Australian*, 02 October 2021). https://www.theaustralian.com.au/nation/police-surround-shrine-of-remembrance-as-antilockdown-protesters-gather/news-story/76121ba8b28c7101f84df767a6d03b9f. Accessed 07 March 2022; Nick Cater, ‘The Tyranny of Ineptitude Drives More to the Streets’ (*Australian*, 23 November 2021). https://www.theaustralian.com.au/commentary/the-tyranny-of-ineptitude-drives-more-to-the-streets/news-story/0e5370a6bd54c95611634dfc92b7038d. Accessed 05 February 2022.

161 Madi Day and Bronwyn Carlson, ‘White Supremacists and Far Right Ideology Underpin Anti-vax Movements’ (*Conversation*, 22 November 2021). https://theconversation.com/white-supremacist-and-far-right-ideology-underpin-anti-vax-movements-172289. Accessed 05 February 2022.

162 ‘Tradie’ is the common Australian abbreviation for ‘tradesman’. The protests were initially a response to the Victorian government decision to impose restrictions on construction sites: Lucy Mae Beers, ‘Melbourne Tradies Stage Protest outside CFMEU Headquarters and Throw Bottles’ (*7 News.com.au*, 20 September 2021). https://7news.com.au/business/workplace-matters/melbourne-tradies-stage-protest-outside-cfmeu-headquarters-and-smash-window-c-4013877. Accessed 07 March 2022. Later protests were characterised by demonstrators wearing high visibility vests characteristic of tradesmen, but there were allegations that these were not real tradesmen: Annika Smethurst, ‘Truth Is More Complex Than Partisan Protest Blame Game’ (*Age*, 22 September 2022). https://www.theage.com.au/politics/victoria/truth-is-more-complex-than-protest-blame-game-20210922-p58tsq.html. Accessed 12 March 2022. The involvement of unionised tradesmen was significant because the Victorian government was broadly aligned with the Union movement.

163 Lidia Kelly, ‘Australia Vaccine-Mandate Protesters Compare State Govt to Nazis—Media’ (*Reuters*, 12 November 2021). https://www.reuters.com/world/asia-pacific/australia-vaccine-mandate-protesters-compare-state-govt-nazis-media-2021-11-13/. Accessed 05 February 2022.

164 Mostafa Rachwani, ‘Melbourne Protests: Prop Gallows Seen as Thousands March against Victoria’s COVID Powers’ (*Guardian*, 13 November 2021). https://www.theguardian.com/australia-news/2021/nov/13/melbourne-protests-prop-gallows-seen-as-thousands-march-against-victorias-covid-powers. Accessed 05 February 2022; Brooke Rolfe, ‘Melbourne Protest: Gallows Spotted at “Kill the Bill” Rally’ (*News.com.au*, 25 November 2021). https://www.news.com.au/technology/online/social/melbourne-kill-the-bill-protest-takes-putrid-turn-at-hands-of-noosewielding-maniacs/news-story/11e46b147219a9ef77bef0514680ca28. Accessed 05 February 2022.

165 Chanel Zagon, ‘Disturbing Protest Scenes of Replica Daniel Andrews on Makeshift Gallows Condemned by Leaders’ (*Nine.com.au*, 16 November 2021). https://www.9news.com.au/national/coronavirus-victoria-update-protests-continue-despite-changes-to-pandemic-legislation/b39f2151-bf96-4d0d-bdcc-e12451a2080b. Accessed 29 November 2021.

166 Connor Perrett, ‘Why Anti-lockdown Protests Are a “Magnet” for White Supremacists and Far-Right Extremists’ (*Business Insider*, 21 May 2020). https://www.businessinsider.in/international/news/why-anti-lockdown-protests-are-a-magnet-for-white-supremacists-and-far-right-extremists/articleshow/75854436.cms. Accessed 05 February 2022.

167 Edward P Thompson, ‘The Moral Economy of the English Crowd in the Eighteenth Century’ (1971) 50 *Past and Present* 76.

168 Calla Wahlquist, ‘Questioning Daniel Andrews: How Reporters Came Under Attack in Victoria’ (*Guardian*, 02 October 2020). https://www.theguardian.com/australia-news/2020/oct/03/questioning-daniel-andrews-how-reporters-came-under-attack-in-victoria. Accessed 05 February 2022.

169 Ibid.

170 Ibid.

171 Victorian Government, ‘Staying Apart Keeps Us Together Video’. https://www.vic.gov.au/media/14574. Accessed 30 November 2021.

172 Australian Government Department of Health, ‘Help Protect Those Most at Risk’. https://m.facebook.com/healthgovau/videos/710685416368653/?locale2=ur_PK. Accessed 29 April 2022.

173 Victorian Government, ‘Fines, Enforcement and Reporting’. https://www.coronavirus.vic.gov.au/fines-enforcement-and-reporting. Accessed 30 November 2021.

174 See, for instance, Norman Hermant, ‘At Melbourne’s Anti-lockdown Protests, Everyone Has a Different Version of the Truth’ (*ABC News*, 26 September 2021). https://www.abc.net.au/news/2021-09-26/anti-lockdown-covid-protests-melbourne-mainstream-media-distrust/100490148. Accessed 05 February 2022.

175 Bleakly, ‘The Fight to Remain Compliant’ (n 157).

176 Ibid.

177 Shahinaz Abdelhamid, ‘Australia’s COVID-19 Response: The Achilles’ Heel’ in Roland Rich (ed), *COVID-19 under Democracy and Autocracy* (State University of New Jersey 2021) 19.

178 Jennifer Coate, *COVID-19 Hotel Quarantine Inquiry* (Victorian Government 2020) vol 1. https://www.quarantineinquiry.vic.gov.au/volume-i. Accessed 20 April 2022.

179 WorkSafe Victoria, ‘Charges Laid over Hotel Quarantine’, Media Release (29 September 2021). https://www.worksafe.vic.gov.au/news/2021-09/charges-laid-over-hotel-quarantine. Accessed 29 November 2021.

180 Lucy Mae Beers and Alex Chapman, ‘Another Melbourne Tradie Protest as Victoria Police and Workers Gather at CFMEU on Tuesday’ (*7 News.com.au*, 21 September 2021). https://7news.com.au/business/workplace-matters/another-melbourne-tradie-protest-as-victoria-police-and-workers-gather-at-cfmeu-on-tuesday-c-4023251. Accessed 05 February 2022.

181 Rob Manwaring and Geoffrey Robinson, ‘What Is “Labor” about Labor State Governments in Australia?’ (2020) 66(1) *Australian Journal of Politics and History* 3, 7.

182 Ibid. 9.

183 Joseph Thwaites, ‘Public Interest in the Pandemic: A Comparative Framing Analysis of COVID-19 Public Health Interventions by the Victorian State Government and Australian Digital News Outlets’ (Master’s Thesis, Lund University 2021) 12.

184 Michel Foucault, *The History of Sexuality* (Robert Hurley tr, Random House 1978) vol 1, 101.

185 Jonah Berger and Katherine L Milkman, ‘What Makes Online Content Viral?” 2012 49(2) *Journal of Marketing Research* 192; Ronald N Jacobs, ‘Virality, Algorithms, and Illiberal Attacks on the Press: Legitimation Strategies for a New World’ (2020) 14(2) *Sociologica* 217.

186 See, for instance, in the context of the United States’ election, Emily Van Duyn and Jessica Collier, ‘Priming and Fake News: The Effects of Elite Discourse on Evaluations of News Media’ (2019) 22(1) *Mass Communication and Society* 29.

187 Gisela Gonçalves, ‘Who’s Afraid of Social Media? A Normative Approach to Corporate Discourse on the Web’ in Gisela Gonçalves (ed). *The Dialogue Imperative: Trends and Challenges in Strategic and Organisational Communication* (LabCom Books 2012).

188 Edward Bernays, ‘The Engineering of Consent’ (1947) 250(1) *Annals of the American Academy of Political and Social Science* 113.

189 Bove, ‘Politics without Romance?’ (n 103) 330.

190 The Public Health and Wellbeing Amendment (Pandemic Management) Act passed after extensive negotiation with independent and cross-bench parliamentarians. Rachel Baxendale, ‘Andrews Government Secures Extra Vote on Pandemic Bill’ (*Australian*, 30 November 2021). https://www.theaustralian.com.au/nation/politics/time-ticking-away-for-stalled-victorian-pandemic-bill/news-story/9dfd0c63db388e84774959fc8f90757f. Accessed 07 March 2022.

191 Simone Fox Koob, ‘Melbourne “Freedom” Protesters Test Positive for COVID-19, One in Hospital’ (*Age*, *29* November 2021). https://www.theage.com.au/national/victoria/melbourne-freedom-protesters-test-positive-for-covid-19-one-in-hospital-20211129-p59d04.html. Accessed 05 February 2022.

192 Baxendale and Varga, ‘Daniel Andrews Defends Proposed Laws Likened to “Stasi Powers” by Lawyers’ (n 3).

193 Human Rights Law Centre, ‘Explainer: Victoria’s Proposed New Pandemic Law’ (10 November 2021). https://www.hrlc.org.au/factsheets/2021/11/10/explainer-victorias-proposed-new-pandemic-law. Accessed 30 November 2021.

194 Eden Gillespie, ‘Debate and Protests Have Been Raging over Victoria’s Pandemic Powers Bill. Here’s Why’ (*SBS News*, 17 November 2022). https://www.sbs.com.au/news/article/debate-and-protests-have-been-raging-over-victorias-pandemic-powers-bill-heres-why/zsibgq5ne. Accessed 07 March 2022; Baxendale, ‘Andrews’ Government Secures Extra Vote on Pandemic Bill’ (n 190).

195 ‘Former Labor MP Adem Somyurek to Block Victorian Pandemic Bill’ (*Sky News*, 18 November 2021). https://www.skynews.com.au/australia-news/coronavirus/former-labor-mp-adem-somyurek-to-block-victorian-pandemic-bill/video/f3cbb057a63f3e9668f18edf06efe5d8. Accessed 12 March 2022.

196 Baxendale, ‘Andrews Government Secures Extra Vote on Pandemic Bill’ (n 190).

